# Effect of Zn Addition on the Cd-Containing Anaerobic Fermentation Process: Biodegradation and Microbial Communities

**DOI:** 10.3390/ijerph16162998

**Published:** 2019-08-20

**Authors:** Yonglan Tian, Huayong Zhang, Lei Zheng, Shusen Li, He Hao, Hai Huang

**Affiliations:** Research Center for Engineering Ecology and Nonlinear Science, North China Electric Power University, Beijing 102206, China

**Keywords:** compound heavy metals, anaerobic fermentation, process stability, substrate biodegradation, enzyme activity, microbial properties

## Abstract

Anaerobic fermentation is considered as a cost-effective way of biomass waste disposal. However, the compound heavy metals contained in the biomass may induce complex effects on anaerobic fermentation, which limit the utilization of metal-contaminated biowaste. In this study, the impacts of Cd and Zn addition on biogas properties, process stability, substrate biodegradation, enzyme activity, and microbial properties were studied. The results showed that the addition of Cd together with Zn (Cd+Zn) increased the maximum daily and cumulative biogas yields, and brought forward the gas production peak compared with the Cd-added group. Taking the whole fermentation process into account, the promotion effects of adding Zn into the Cd-containing fermentation system on biogas yields were mainly attributable to better process stability, higher average NH_4_^+^-N concentration in the later stage of fermentation, reduced COD (*p* < 0.05), and increased biodegradability of lignocelluloses (*p* < 0.01), especially cellulose (*p* < 0.05) and lignin (*p* < 0.01). Meanwhile, the addition of Zn promoted the coenzyme M activity (*p* < 0.05), and increased the absolute abundance of *Methanothermobacter*. The bacteria communities during the fermentation process were responsible for the degradation of lignocelluloses. The results demonstrated that the addition of appropriate Zn into the Cd-containing fermentation system enhanced the efficiency of anaerobic fermentation and utilization of biowaste.

## 1. Introduction

With the rapid development of industries such as metal plating facilities, mining operations, fertilizer industries, tanneries, batteries, paper industries, pesticides, etc., large areas of land and water are polluted by heavy metals. For soil pollution, phytoremediation shows great potential in remediating heavy metal contamination via the enrichment and hyperaccumulation of specific heavy metals, transferring heavy metals from the soil to plants [1,2]. During the process of phytoremediation, the rapid growth of plants produces a large amount of lignocellulosic biomass, which can be used as an energy resource by technologies, such as anaerobic fermentation [3,4].

In recent years, lignocellulosic biomass containing heavy metals were utilized as raw materials for anaerobic fermentation [5,6], which expressed different degrading patterns compared with the single feedstock fermentation. The anaerobic degradation process of complex organic compounds has been previously reported [7]. In general, the fermentation process with animal manure as a single feedstock would express a one-phase decomposition [8,9]. However, the anaerobic fermentation process with mixed lignocellulosic biomass and animal manure and other inoculums was likely to show a diauxie growth process [1,8]. It was caused by the complexity of the lignocellulosic biomass degradation. Moreover, previous studies showed that heavy metals promoted biogas production within a certain concentration range. The addition of metals, in particular, compound metals would accelerate the degradation of lignocellulosic biomass and induce the one-phase decomposition [10]. 

During the process of anaerobic fermentation, the core pathways that generate biogas are enzymes catalyzing redox reactions. Many of these enzymes contain transition metals as cofactors for electron transport or as catalytic centers at active sites [11,12]. The metallo-enzymes present in different microorganisms and the metals that are essential for these enzymes have been previously summarized by Zandvoort et al. [7]. Hydrolases like cellulase, methanogenesis involved enzymes like coenzyme F_420_ can be promoted by adding certain metals [10]. Therefore, metals are of importance for fermentation microorganisms’ optimal growth and performance [13]. 

Cd contamination is a worldwide environmental and health concern [14]. Cd would enter the anaerobic fermenters along with the biomass. The impacts of Cd on the anaerobic fermentation were studied in the past decades and they were found to promote the biogas production in certain concentrations [1,15,16,17,18]. A study on biogas production with maize contaminated with Cd as feedstocks (the final Cd concentration of residues achieved 5.34 mg/kg) found no inhibitory effects during the anaerobic digestion process [19]. Cd concentrations of 2.00 ± 0.44, 39.80 ± 1.25, and 6.37 ± 0.15 mg/kg in the shoot of canola, oat, and wheat improved the biogas yields by 59.37%, 79.23%, and 11.34% than the control group [1]. Elizabeth found that Cd^2+^ activated the methanogenesis in the marine archaeon *Methanosarcina acetivorans* [20]. With acetate as substrate, Cd^2+^ slightly increased both *Methanosarcina acetivorans* growth and CH_4_ rate synthesis. 

Zn is widely distributed in plant, soil, and water [21] and often appears together with Cd during mining. Zn takes part in the functioning of enzymes involved in methanogenesis such as coenzyme M methyltransferase [22]. Bożym found that the addition of Zn in the anaerobic fermentation system weakened the toxicity of Cd and increased biogas production [23]. However, to the best of our knowledge, the impacts of the mechanisms of compound Cd and Zn on the anaerobic fermentation process has not been extensively studied, which has hindered the efficient utilization of biowaste.

The objective of this study is to explore the effect of Zn addition on the Cd-containing anaerobic fermentation process. The effects of Cd and Cd+Zn addition on thermophilic anaerobic fermentation process with mixed corn stover and cow manure as feedstocks were studied. The biogas properties, process stability, substrate degradation, and enzyme activity, as well as the microorganisms, particularly methanogens, during the fermentation process, were studied. This study is expected to provide valuable information for mechanism research of compound metals on anaerobic fermentation and on the utilization of heavy metal contaminated biowaste.

## 2. Materials and Methods

### 2.1. Experimental Materials

The corn stover, a representation of lignocellulosic biomass, was used as the feedstock in the present research. The corn stover was collected from the farmland in Tongzhou District, Beijing in November 2016. When harvested, 10 cm corn stover above the ground was left in the field. The harvested corn stover was cut into pieces of 5 to 10 cm in length, and air-dried until moisture levels reached <10%. The dried stover was then ground into a powder and passed through a 10 mesh sieve. Fresh cow manure was used as an inoculum and was collected from the Yanqing base, Beijing Dairy Cattle Centre. The fresh cow manure was stored at 4.0 °C after being collected. The properties of the corn stover and the cow manure are shown in Table 1. 

### 2.2. Anaerobic Fermentation Experiment

**Figure 1:**
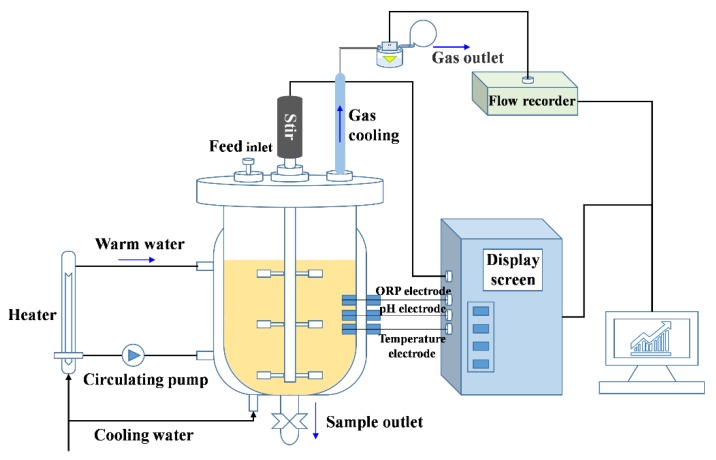
The fermentation system used for anaerobic fermentation experiments.

The experiments were performed in the anaerobic fermenters (30 L total volume and 20 L available volume, YGF 300/30, Shanghai Yangge Biological Engineering Equipment Co., Ltd., Shanghai, China) for 28 days at 55 ± 1.0 °C (automatic controlled as shown in Figure 1) as previously reported [24]. The fermenters were autoclaved before feeding. Equal amounts of corn stover and cow manure (dry weight 0.8 kg of each) were mixed as the substrates for fermentation. The total solids (TS) of the substrate in the reactors were adjusted to 8% by adding distilled water.

At the beginning of fermentation, 0.041 g CdCl_2_·5/2H_2_O was added into two fermenters with the calculated concentrations of 1.0 mg/L. Synchronously, 2.0 mg/L Zn (0.083 g ZnCl_2_) was added to one of the fermenters. Then, the reactors were purged with N_2_ gas for 5 min to remove oxygen in the digestion system. The contents in the fermenter were thoroughly stirred by a three-layer stirrer introduced in the middle of the reactor for 30 min every morning from 8:30 a.m. to 9:00 a.m. (Figure 1). The initial pH values were not adjusted before experiments.

### 2.3. Measurements

Biogas yields, pH values, and oxidation-reduction potential (ORP) were automatic measured at 9:00 a.m. every day [21]. Solid, liquid, and gas samples were collected every three days at 9:00 a.m. Total solid (TS) was measured by weighing the samples after drying at 105 °C in drying cabinet (GZX-9030 MBE, Shanghai Boxun Industrial Co., Ltd., Shanghai, China) for 24 h [25]. Volatile solid (VS) was measured after treating the samples in a muffle at 550 °C, for 1 h [25]. Total nitrogen (TN) was measured by Indophenol blue colorimetric method after being digested by concentrated sulfuric acid and 30% hydrogen peroxide [17]. Total organic carbon (TOC) was measured by potassium dichromate volumetric method [26]. Cellulose, hemicellulose, and lignin in solid were determined by cellulose, hemicellulose, and lignin Enzyme-Linked Immunosorbent Assays kit (ELISA, Qingdao Kebiao Testing and Research Institute Co. LTD, Qingdao, China), respectively [10]. NH_4_^+^-N was measured by Nessler’s reagent method [27]. Chemical oxygen demand (COD) in the supernatant was obtained by the potassium dichromate method after sample centrifugation at 5000 rpm for 10 min [28]. The cellulase, coenzyme M, and coenzyme F_420_ activities in the supernatant were determined according to the standard method after centrifugation at 4000 rpm for 5 min [29]. CH_4_ contents in biogas were measured by a gas chromatograph (GC–2014C, Shimadzu Co., Kyoto, Japan) equipped with a GDX–401 column with H_2_ as the carrier gas. Detection was performed with a thermal conductivity detector (TCD) [22]. 

The measurement of microbial communities was conducted by Novogene Co., Ltd. (Beijing, China) after certificating the samples at 8000 rpm and 4 °C for 3 min. Briefly, the genomic DNA of the samples on the 7th, 13th, and 19th day were extracted by cetyltrimethylammonium ammonium bromide (CTAB) methods [27]. After the extraction, the samples were diluted to a concentration of 1 ng/L with sterile water. Then the diluted genomic DNA was used as the template for PCR amplification. PCR amplification of the V3–V4 hypervariable region of bacterial 16S rDNA was performed using universal primers 338F (50-ACTCCTACGGGAGGCAGCAG-30) and 806R (50-GGACTACHVGGGTWTCTAAT-30) [26]. Archaea primers used to amplify the V3–V4 hypervariable region of archaeal 16S rDNA were 344F (50-ACGGGGYGCAGCAGGCGCGA-30) and 806R (50-GGACTACVSGGGTATCTAAT-30). All primers included Illumina barcode sequences for multiplexing each sample. The library construction was conducted with TruSeq^®^ DNA PCR-Free Sample Preparation Kit. After the Qubit and Q-PCR quantification, the constructed library was qualified and HiSeq2500 PE250 was used for sequencing.

### 2.4. Data Analysis

After removing the barcode and primers’ sequences, the reads were matched with FLASH (V1.2.7) for raw tags and then sieved for clean tags. Clean tags were cut out and the length filtered by referencing the Qiime quality control process (V1.9.1). The obtained tags were treated by removing the chimeric sequence through comparison with the detection chimeric sequence (Gold database) yielding the final effective tags as the targets. The cluster analysis of effective tags was conducted using Uparse software (V7.0.1001). The operational taxonomic units (OTUs) were clustered with an identity >97%. Species annotation of the OTUs representative sequence was carried out using Mothur method and SSUrRNA database (define the threshold of 0.8–1.0). The microbial communities were then obtained after annotation. 

The data in the study were the average of three repeats. Error bars represent the standard errors of the mean: SEM=SD/$\sqrt{n}$, where SD is the standard deviation. Paired-sample *t*-test and Pearson correlation analysis were performed in Statistical Package for the Social Science (SPSS, 17.0, Chicago, IL, USA) software at 0.05 and 0.01 levels of significance by * (*p* < 0.05) and ** (*p* < 0.01), respectively.

## 3. Results and Discussion

### 3.1. Impact of Cd and Zn Addition on Biogas Production

#### 3.1.1. Biogas Yields

**Figure 2:**
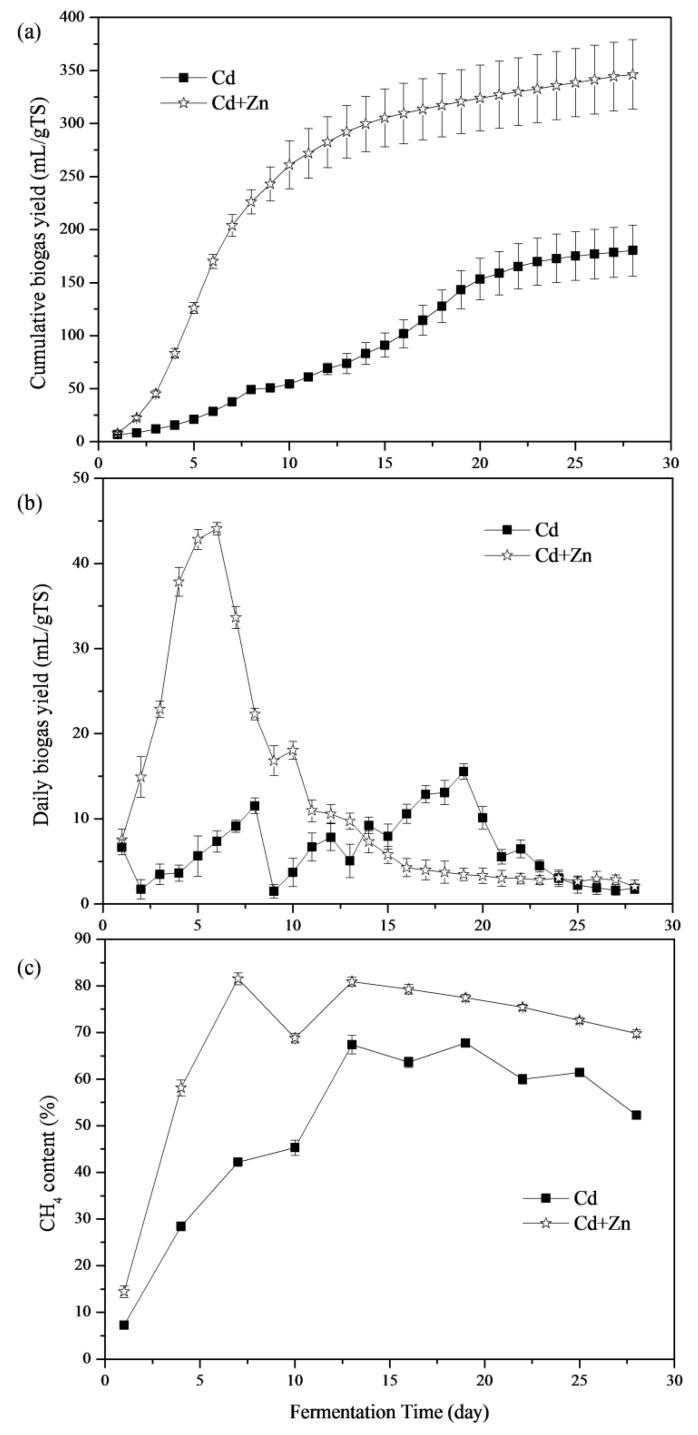
Cumulative biogas yields (**a**), daily biogas yields (**b**), and CH_4_ contents (**c**) in response to Cd and Cd+Zn during the fermentation.

The impacts of Cd and Cd+Zn addition on biogas production are shown in Figure 2a. The Cd+Zn-added group produced more biogas than the Cd-added group throughout the fermentation process. The cumulative biogas yields of the Cd+Zn-added group was 1.92 times higher than that of the Cd-added group at the end of the experiment. Thus, combining Cd with Zn promoted biogas production.

The daily biogas yields throughout the fermentation process are depicted in Figure 2b. The maximum daily biogas yield, 44.09 mL/g TS, was obtained in the Cd+Zn-added group, which was 2.83 times higher than that of Cd-added group. The Cd+Zn-added group reached the biogas peak on the 6th day, which was 13 days earlier than the Cd-added group. Thus, adding Zn into the Cd-containing anaerobic fermentation system was able to improve and bring forward the daily biogas peak. There were two daily biogas peaks in the Cd-added group. According to previous research [21], the first peak of gas production was due to the degradation of cow manure, and the second peak may be caused by the degradation of corn stover. However, there was only one daily biogas peak in the Cd+Zn-added group, which may be due to the addition Zn into the Cd-containing anaerobic fermentation system bring forward the second daily biogas peak, making the two daily biogas peaks coincide.

#### 3.1.2. CH_4_ Content

CH_4_ production is considered as the performance indicator during the anaerobic process [30]. Figure 2c illustrates the CH_4_ content of the biogas in different groups. The CH_4_ content of the Cd+Zn-added group was significantly higher than that of the Cd-added group during the whole fermentation process. On the 1st day, the CH_4_ content and the biogas yield for Cd-added and Cd+Zn-added groups were low. Then, the CH_4_ contents presented an obviously up-trend. After the 7th day, the CH_4_ content of the Cd+Zn-added remained stable at around 70% while the Cd-added group continued to increase until the 13th day. The CH_4_ contents of the Cd+Zn-added group were significantly higher than that of the Cd-added group (*p* < 0.05, *t*-test). CH_4_ contents in the Cd-added and Cd+Zn-added groups were higher than the previous study with cow manure-based waste mixtures as feedstocks [30]. Therefore, the addition of Cd improved CH_4_ production and the further addition of Zn enhanced the stimulatory effect. 

### 3.2. Process Stability

#### 3.2.1. Variations of pH Values

**Figure 3:**
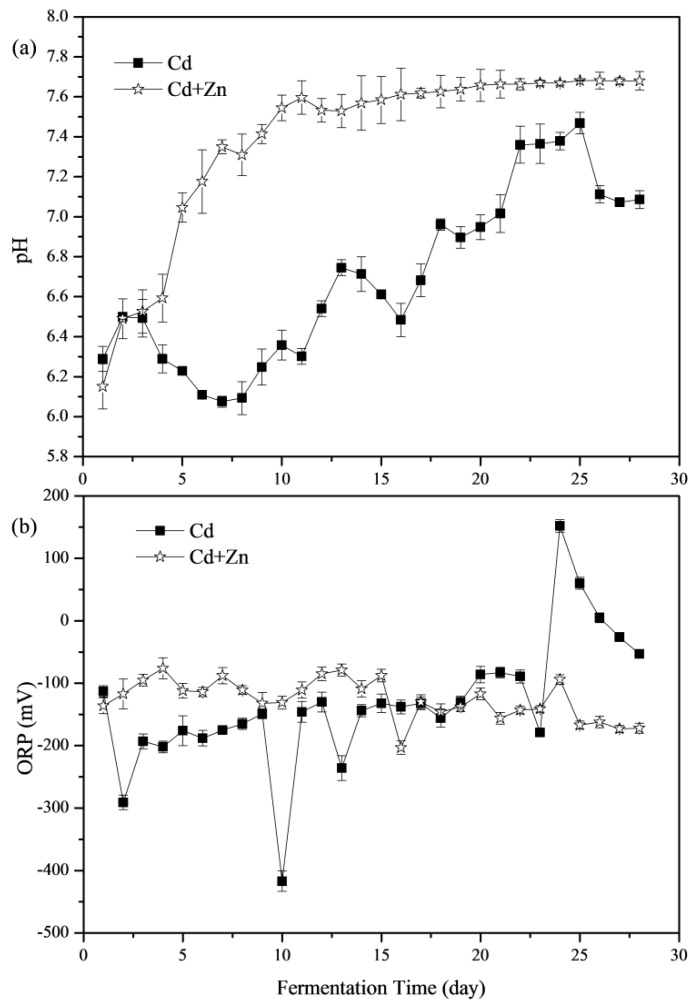
Impact of Cd and Cd+Zn addition on pH values (**a**) and oxidation-reduction potential (ORP) (**b**) during the fermentation.

The importance of pH values in representing the status of fermenters and impacting the activity of microbial communities has been widely concerned [8,31,32]. The variations of pH values during the fermentation process are shown in Figure 3a. During the whole fermentation process, the average pH values of the Cd and Cd+Zn groups were 6.69 and 7.39, respectively. The pH values of the Cd+Zn-added group were significantly higher than that of the Cd-added group (*p* < 0.05, *t*-test). The pH values of both groups were located in the optimal pH range (6.4–7.4) for anaerobic microorganisms [33]. In the previous study, during the start-up stage of fermentation, the organic components in the substrate were quickly hydrolyzed into acids, which were not efficiently used for methanogenesis due to the slow adaption and metabolism of methanogens. Thus, acidic hydrolytic products accumulated, resulting in a decrease in pH values and low biogas yield [34]. However, for the Cd+Zn-added group, the pH rose from the beginning and increased greatly from the 4th day and then stabilized, which were different from a previous study [21]. It was because that the acidic components hydrolyzed from organic materials in feedstocks were efficiently used for methanogenesis and thus were not accumulated in this experiment.

From the 14th day, the pH of the Cd+Zn-added group became stable, while the Cd-added group experienced a larger pH variation. Thus, the addition of Zn in the Cd-containing fermentation system benefited the stability of fermentation process and enhanced the acid-base buffering capacity of the fermentation system.

#### 3.2.2. Oxidation-Reduction Potential (ORP)

The ORP value is an indicator of the redox state of the fermentation system, and is also an important parameter for controlling the anaerobic fermentation system, and the lower the ORP value, the deeper the anaerobic level of the fermentation system [35]. Figure 3b illustrates the variation of ORP values at different groups during the anaerobic fermentation. According to the *t*-test, there was no significant difference between the ORP values of Cd-added and Cd+Zn-added groups (*p* > 0.05). The ORP values in the Cd+Zn-added group were relatively stable throughout the fermentation process; however, the ORP values in the Cd-added group fluctuated considerably. In addition, the daily biogas yields and the CH_4_ contents in the Cd+Zn-added group were relatively high. It manifested that the stable redox state in the Cd+Zn-added group during the fermentation process played an important role in maintaining the stability of the fermentation process and ensuring the higher biogas production efficiency than the Cd-added group. 

### 3.3. Substrate Biodegradation

#### 3.3.1. COD

**Figure 4:**
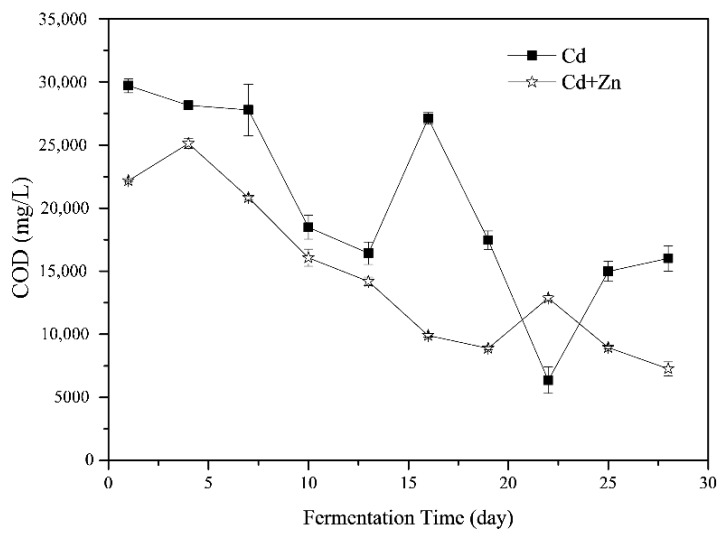
Impact of Cd and Cd+Zn addition on chemical oxygen demand (COD) during the fermentation.

The variations of COD during the fermentation process are shown in Figure 4. The COD in the fermenter was the result of the balance of hydrolysis, acidification, acetogenesis and methanogenesis stages [36]. The average COD of the Cd-added was 20241.72 ± 2401.54 mg/L which was significantly higher than that of the Cd+Zn-added groups (14627.23 ± 1977.83 mg/L, *p* < 0.05, *t*-test). In general, the COD in the Cd+Zn-added group increased first and then decreased, while the COD in the Cd-added group decreased first and then rose and finally declined (Figure 4). From the 4th to the 10th day of the fermentation, the COD in the Cd+Zn- group decreased significantly, corresponding to the increase of cumulative and daily biogas yields. This result indicated that organic components in the liquid phase were efficiently utilized for biogas production. After the 19th day, the COD was low in both experimental groups. Corresponding to the low daily gas production of the two groups, the substances that be converted to CH_4_ by the methanogens was insufficient during this period. In other words, the main factor limiting anaerobic fermentation at this time was the insufficient supply of fermentation substrate.

#### 3.3.2. NH_4_^+^-N Concentrations

**Figure 5:**
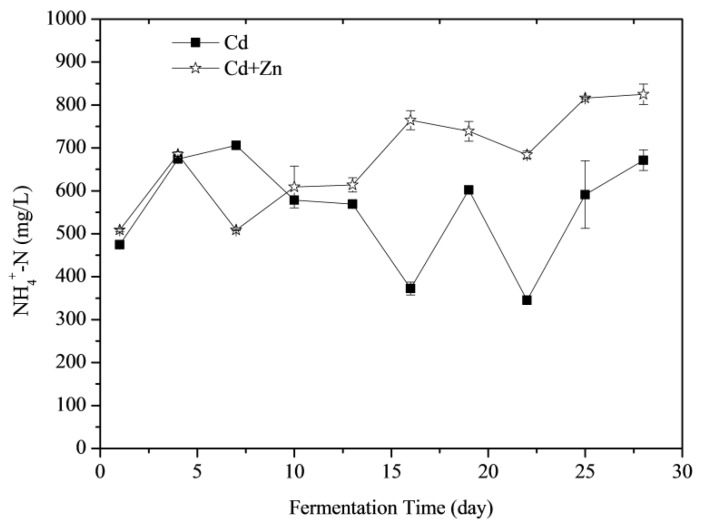
Impact of Cd and Cd+Zn addition on ammonia nitrogen (NH_4_^+^-N) concentrations during the fermentation.

NH_4_^+^-N was able to increase the activity of methanogenic bacteria as an important source of nitrogen whilst increasing the alkalinity of the anaerobic digestion at a lower concentration in the fermentation reaction system and improving the cushioning properties of the system to the volatile organic acid [37]. Effects of Cd and Zn addition on NH_4_^+^-N concentrations during the fermentation were shown in Figure 5. According to the *t*-test, there was no significant difference between the NH_4_^+^-N concentrations of Cd-added and Cd+Zn-added groups in the view of the whole fermentation process (*p* > 0.05). However, adding Zn induced different performance of NH_4_^+^-N concentrations at different stages of fermentation. Before the 4th day of fermentation, the NH_4_^+^-N concentrations of the two groups increased because the organic nitrogen in the fermentation substrate was converted to NH_4_^+^-N during the degradation process. From the 4th day to the 7th day, the NH_4_^+^-N of the Cd+Zn-added group decreased corresponding with the daily biogas peak, indicating that methanogens in this group consumed more nitrogen for biogas production. After the 10th day, the NH_4_^+^-N concentrations in the Cd-added group changed greatly, while the NH_4_^+^-N concentrations in the Cd+Zn-added group were relatively stable. It indicated that the addition of Zn into the Cd-containing fermentation system enhanced the stability of the NH_4_^+^-N concentration, created a favorable living environment for methanogens and thereby producing more gas.

#### 3.3.3. Lignocellulose Contents

**Figure 6:**
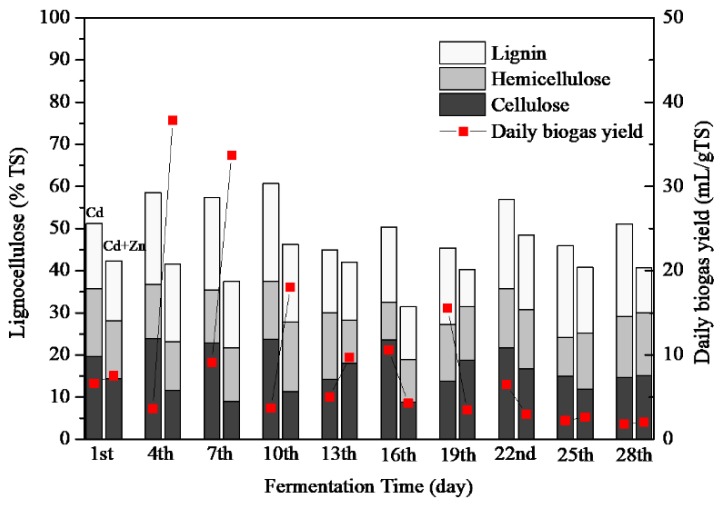
Impact of Cd and Cd+Zn addition on lignocellulose contents during the fermentation.

Lignocelluloses are mainly composed of cellulose, hemicellulose, lignin [18]. Although cellulose and hemicellulose were easily decomposed by microorganisms when they are present alone, their biodegradability is greatly reduced when they are present as lignocellulose complexes [38]. The lignocellulose concentrations of Cd-added and Cd+Zn-added groups are presented in Figure 6. The average lignocellulose contents were 52.25 ± 3.07% and 39.36 ± 2.84% for the Cd-added and Cd+Zn-added groups, respectively. Zn addition significantly enhanced the degradation of lignocelluloses (*p* < 0.01).

The average content of lignin, hemicellulose, and cellulose in the Cd+Zn-added group was lower than that in the Cd-added group (Table 2). Therefore, the addition of Cd+Zn was conducive to destroy the structure of lignocellulose, and provide hydrolytic products for further fermentation. Nevertheless, there was no obvious relationship between the composition of lignocellulose and the biogas yield, which required further study in the future. 

### 3.4. Effect of Cd-Containing Compound Pollution on Enzyme Activity

#### 3.4.1. Cellulase

**Figure 7:**
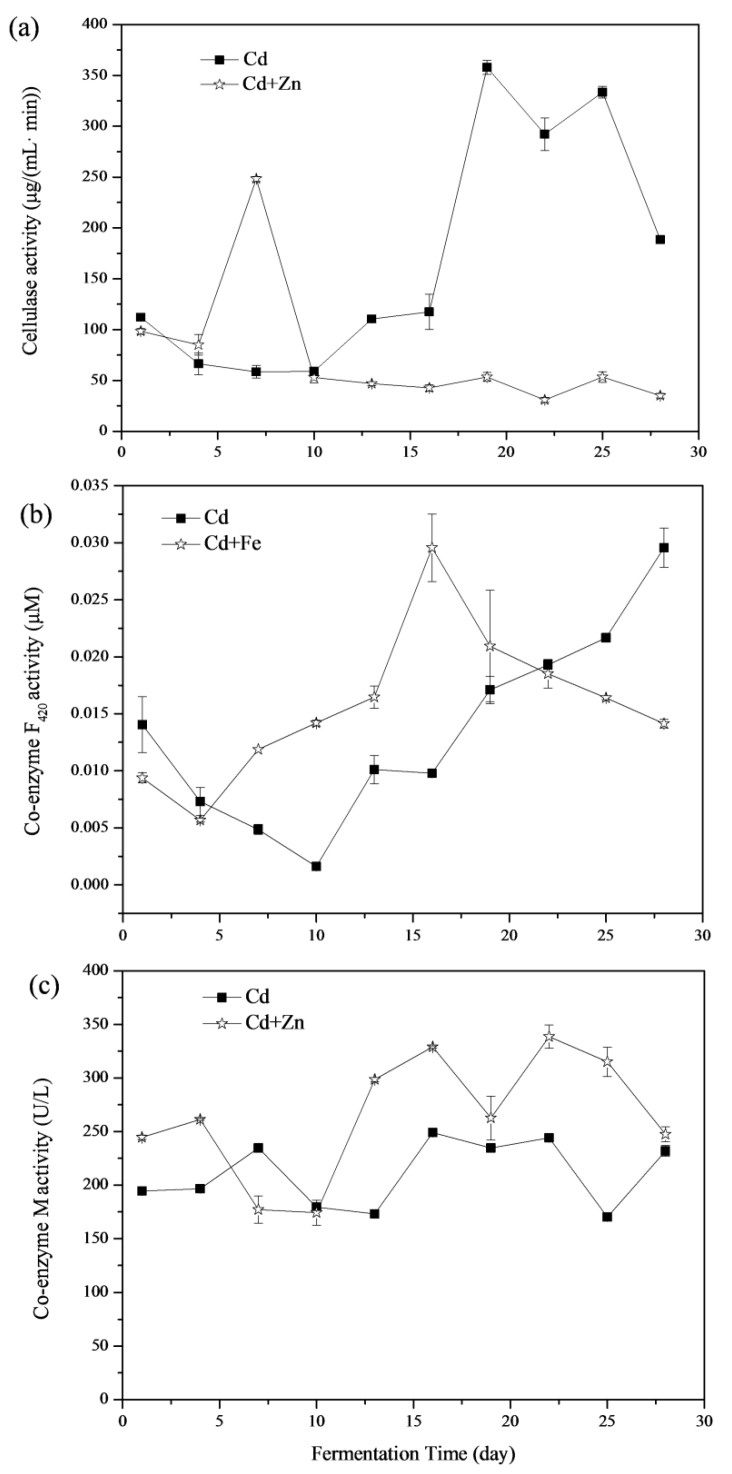
Impact of Cd and Cd+Zn addition on cellulase activity (**a**) coenzyme F_420_ activity, (**b**) coenzyme M, and (**c**) activity during the fermentation.

Enzymatic hydrolysis of cellulose is carried out by cellulase enzymes, which are highly specific [39]. Cellulase degrades cellulose and releases reducing sugars as the end products [40]. The variations of cellulase activities are shown in Figure 7a. The average cellulase activity in the Cd and Cd+Zn groups was 169.58 ± 7.49 and 74.66 ± 4.84 μg/(mL min). There was no significant difference between these two groups in the view of the whole fermentation process (*p* > 0.05, *t*-test). From the 4th to the 7th day of the fermentation, the cellulase activities of the Cd+Zn-added group were higher than that of the Cd-added group and reached the maximum on the 7th day. At this time, the biogas production in Cd+Zn-added group was consistently higher than that in Cd-added group. However, after the 10th day, cellulase activity in the Cd+Zn-added group remained at a low level and was much lower than that in the Cd-added group (*p* < 0.05, *t*-test), and the daily biogas production in the Cd-added group exceeded that in the Cd+Zn-added group on the 14th day. During this period, the cumulative biogas yields and CH_4_ contents in the Cd+Zn-added groups were consistently higher than that in the control group, which indicated that the non-cellulosic material in the substrate was the main contributor to the biogas yield. 

#### 3.4.2. Coenzyme F_420_

The low-potential electron carrier, coenzyme F_420_, is an 8-hydroxy-5-deazaflavin presenting in methanogenic bacteria [41]. It plays an important role in the formation of CH_4_ and can be used to reflect the activity of methanogenic bacteria in anaerobic fermentation [42,43]. Variations of coenzyme F_420_ activities were shown in Figure 7b In view of the whole fermentation process, there was no significant difference between the coenzyme F_420_ activity of the Cd-added and Cd+Zn-added groups (*p* > 0.05, *t*-test). The coenzyme F_420_ activity in Cd-added group firstly decreased during the first 10 days and then continuously rose afterward. Differently, the coenzyme F_420_ activity in the Cd+Zn-added group decreased briefly in the first 4 days and then rose the maximum point on the 16th day. This result showed that the addition of Cd+Zn improved the activity of coenzyme F_420_ and then promoted gas production in the first 10 days (Figure 2b). The coenzyme F_420_ activity of the Cd-added group was lower than the Cd+Zn-added group before the 22nd day, and the pH value was low at this stage (Figure 3a). This may be due to the accumulation of acidic components leading to a decrease in the activity of coenzyme F_420_ [21].

#### 3.4.3. Coenzyme M

Coenzyme M is a coenzyme transferred as methyl in the methyl transfer chain, which reflects the number and activity of methanogens [44]. Variations of coenzyme M activities are shown in Figure 7c. The average coenzyme M activity in the Cd and Cd+Zn-added groups were 210.74 ± 3.28 and 264.82 ± 17.67 U/L, respectively. Thus, the coenzyme M activity in the Cd+Zn-added group was significantly higher than in the Cd-added group (*p* < 0.05, *t*-test).

After the 13th day, the coenzyme M activity of the Cd+Zn-added group was continuously higher than in the Cd-added group, whist the CH_4_ content was high as well (Figure 2c). It indicated that the addition of Zn into the Cd-containing fermentation system promoted the activity of methanogens and the coenzyme M activity, resulting in an increase of CH_4_ content. However, for the Cd-added group, the CH_4_ content did not coincide with the change of coenzyme M activity, indicating that the final CH_4_ production was related to other factors.

### 3.5. Microbial Properties

#### 3.5.1. Structure of Bacterial Communities

**Figure 8:**
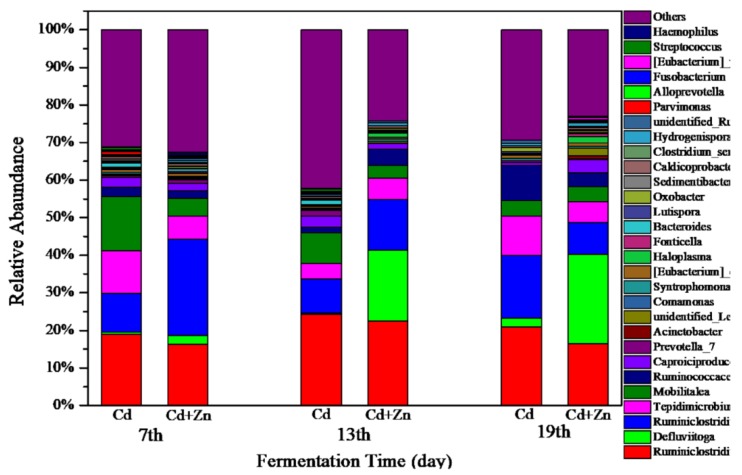
Impact of Cd and Cd+Zn addition on Structure of bacterial communities during fermentation.

The variations of bacterial communities annotated on the level of genus in Cd and Cd+Zn groups on the 7th, 13th, and 19th day of fermentation are shown in Figure 8. In the Cd-added group, the dominant bacterial on the 7th, 13th, and 19th day were *Ruminiclostridium* and *Ruminiclostridium_1*. *Ruminiclostridium* produces extracellular multi-enzymatic complexes called cellulosomes, which efficiently degraded the crystalline cellulose and the cell wall [45]. On the 7th day, the main bacterial was *Mobilitalea*. As previously reported, *Mobilitalea* was able to ferment a variety of mono-, di-, and polysaccharides, including microcrystalline cellulose [45]. The dominant bacteria on the 7th and 19th day was *Tepidimicrobium. Tepidimicrobium* degrades proteins into acetic acid, ethanol, hydrogen, and carbon dioxide [46]. On the 19th day, the main bacterial in the Cd-added group was *Ruminococcaceae_UCG-010. Ruminococcaceae* belongs to the *Firmicutes* and degrades starch, cellulose, other polysaccharides, proteins, and short-chain organic acids, and is an important bacterial family in the hydrolysis stage of anaerobic fermentation [47,48]. Thus, *Ruminococcaceae_UCG-010* in the Cd-added group contributed to the decrease of cellulose on the 19th day (comparing with the cellulose contents on the other days in Figure 5).

In the Cd+Zn-added group, the dominant bacteria on the 7th, 13th, and 19th day were *Ruminiclostridium, Ruminiclostridium_1*, and *Tepidimicrobium*. On the 13th day and 19th day, the abundance of *Defluviitoga* increased. A new isolate L3 of *Defluviitoga tunisiensis* presumably was able to degrade cellulose since genes encoding non-cellulosomal cellulases were identified in its genome [49]. Acetate, H_2_, and CO_2_ were supposed to be end products of the fermentation process. In general, the total relative abundance of *Ruminiclostridium*, *Ruminiclostridium_1*, and *Defluviitoga* in the Cd+Zn-added group was higher than the Cd-added group, resulting in a lower average content of cellulose in the Cd+Zn-added group than that in the Cd-added group. These results indicated that the addition of Zn into the Cd-containing fermentation system was beneficial to the destruction of the cellulose structure, thereby providing hydrolytic products for further fermentation.

#### 3.5.2. Methanogens and Their Relationships with Fermentation Parameters

**Figure 9:**
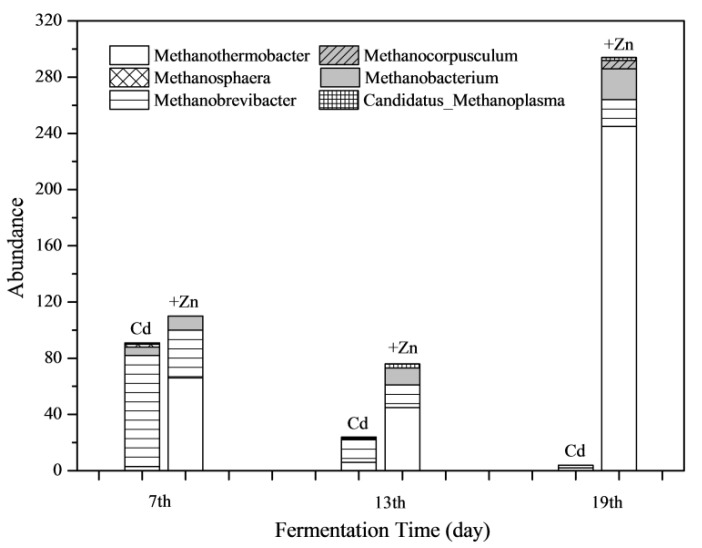
Impact of Cd and Cd+Zn addition on methanogen community composition during the fermentation.

The absolute abundance of methanogens in both Cd and Cd+Zn group at different stages of fermentation are shown in Figure 9. It was found that Zn addition increased the total abundance of methanogens greatly during the whole fermentation process. Especially on the 19th day, the abundance of methanogens in the Cd+Zn-added group was 73.5 times that of the Cd-added group. In view of the whole fermentation process, the proportion of *Methanobrevibacter* in the Cd-added group was the highest. Meanwhile, the abundance of *Methanothermobacter* was highest in the Cd+Zn-added group.

In the anaerobic fermentation system, both *Methanobrevibacter* and *Methanothermobacter* use H_2_ and CO_2_ to produce CH_4_. Methanogens were sensitive to toxic substances, and those methanogens using acetate were more susceptible to toxic substances than methanogens using H_2_/CO_2_ [50]. The *Methanothermobacter* uses NH_4_^+^-N as nitrogen source [51]. The NH_4_^+^-N content in the Cd-added group did not decrease continuously (Figure 5), indicating that the decrease in the abundance of *Methanothermobacter* was not due to the deficiency of nitrogen source.

**Figure 10:**
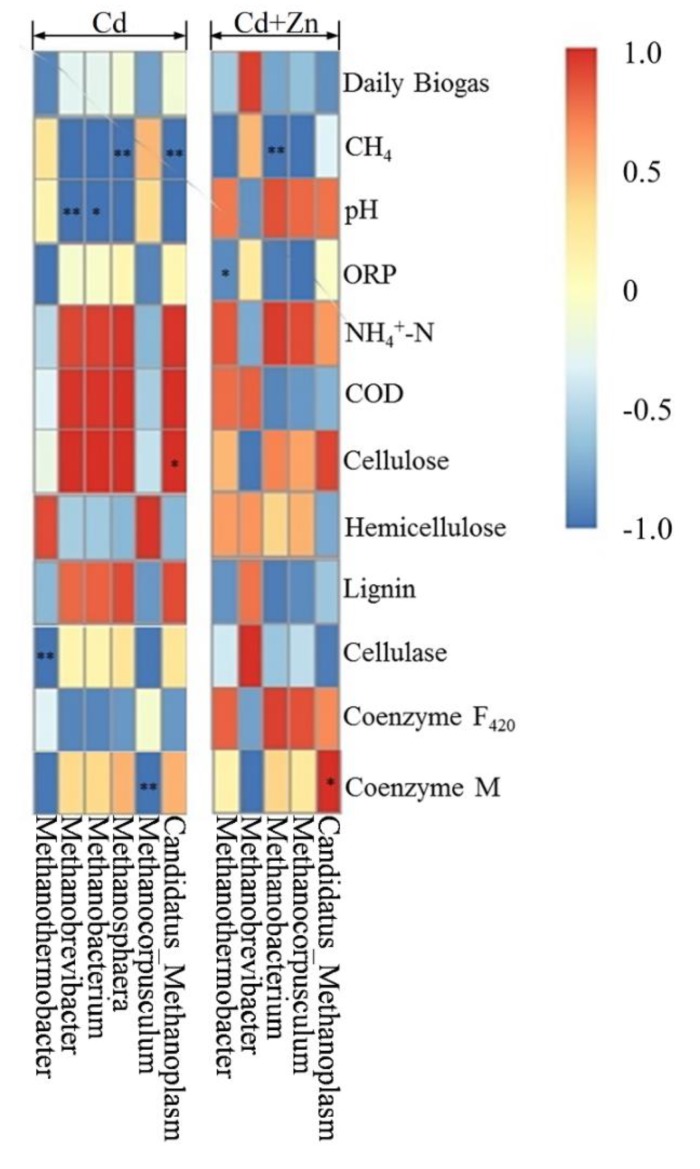
Pearson correlation analysis between environmental factors and methanogen under Cd and Cd+Zn addition during the fermentation.

The Pearson correlation analysis was used to study the relationship between environmental factors and methanogens as shown by the heat analysis results of the correlation in Figure 10. The vertical direction is the information of different environmental factors, and the horizontal indicates the methanogen information. In Cd-added group, *Methanobrevibacter* (*p* < 0.01) and *Methanobacterium* (*p* < 0.05) were significantly negatively correlated to the pH values. However, pH was not correlated to methanogens in the Cd+Zn group. Meanwhile, *Methanosphaera* (*p* < 0.01) and *Candidatus_Methanoplasm* (*p* < 0.01) were significantly negatively correlated to the CH_4_ contents. *Methanothermobacter* was significantly negatively correlated to the cellulose contents (*p* < 0.01) while *Candidatus_Methanoplasm* was positively correlated to the cellulose contents (*p* < 0.05). *Methanobacterium* was negatively correlated to the coenzyme M (*p* < 0.01).

In Cd+Zn-added group, *Methanothermobacter* was significantly negatively correlated to the ORP values (*p* < 0.05). *Methanobacterium* was negatively correlated to the CH_4_ content (*p* < 0.01). Meanwhile, it is worth noting that *Candidatus_Methanoplasma* was positively correlated to the coenzyme M activities (*p* < 0.05). On the other hand, no other detected methanogens were correlated to NH_4_^+^-N, COD, hemicellulose lignin, or coenzyme F_420_ in both Cd and Cd+Zn groups.

According to these results, it was found that the addition of Zn into the Cd-containing fermentation process induced the variation of methanogens during the fermentation process. The variations of methanogens during the fermentation process were responsible for the higher CH_4_ contents in the Cd+Zn-added group than the Cd-added group. However, relationships between the methanogens and the fermentation parameters were not able to explain the impacts of metals on biogas production. It was thus suggested that bacterial community, together with archaeal communities, and, in particular, methanogens, should be determined for analyzing the degradation of the substrate.

## 4. Conclusions

This research studied the different effects of Cd and Cd+Zn addition on anaerobic fermentation with mixed corn stover and cow manure as feedstocks. The addition of Cd together with Zn increased the maximum daily gas yields (92.15%) and cumulative gas yields (183.83%), and brought forward the gas production peak (13 days earlier) compared to the Cd-added group. Adding Zn into the Cd-containing fermentation system was conducive to the stability of ORP and the acid-base buffering capacity of the fermentation system. Zn addition increased the average NH_4_^+^-N concentrations and stability in the later stage of the fermentation, and significantly reduced the COD during the whole fermentation process (*p* < 0.05). The combination of Zn with Cd also promoted the degradation of lignocellulose (*p* < 0.01), especially cellulose (*p* < 0.05) and lignin (*p* < 0.01), which was closely associated with the bacterial communities during the fermentation process. The addition of Cd+Zn increased the absolute abundance of *Methanothermobacter*, decreased the activity of cellulase in the later stage of fermentation, and promoted the activity of coenzyme F_420_ in the middle stage of fermentation and the activity of coenzyme M during the whole fermentation process (*p* < 0.05). The results demonstrated that the addition of appropriate Zn into the Cd-containing fermentation system was able to enhance the efficiency of anaerobic fermentation and utilization of biowaste.

## Figures and Tables

**Table 1:** Characteristics of corn stover and cow manure.

Characteristics	Corn Stover	Fresh Cow Manure
TS (% dry weight)	95.59 ± 0.23	16.49 ± 0.16
VS (%TS)	90.72 ± 0.24	84.00 ± 0.48
TN (%TS)	1.21 ± 0.03	3.22 ± 0.11
TOC (%TS)	13.94 ± 0.64	14.81 ± 0.37
C/N	11.52 ± 0.05	4.45 ± 0.30
Cellulose (%TS)	20.19 ± 1.24	23.56 ± 1.47
Hemicellulose (%TS)	14.05 ± 2.25	16.41 ± 0.48
Lignin (%TS)	13.55 ± 0.07	15.41 ± 1.11
Cr (μg/g dry weight)	8.33 ± 1.13	3.01 ± 0.63
Co (μg/g dry weight)	0.34 ± 0.24	0.61 ± 0.03
Fe (μg/g dry weight)	520.80 ± 67.03	610.80 ± 12.87
Ni (μg/g dry weight)	1.71 ± 0.37	1.78 ± 0.10
Cd (μg/g dry weight)	Negligible	Negligible
Zn (μg/g dry weight)	14.89 ± 1.61	152.44 ± 2.06

Mean ± Standard Error. n = 3. TS, total solid; VS, volatile solid; TN, total nitrogen; TOC, total organic carbon; C/N, ratio of carbon to nitrogen.

**Table 2:** Average contents of cellulose, hemicellulose, lignin, and total lignocellulose during the fermentation.

Group	Lignin (% TS)	Hemicellulose (% TS)	Cellulose (% TS)	Total Lignocellulose (% TS)
Cd	19.27 ± 1.38	13.14 ± 0.75	19.84 ± 0.94	52.25 ± 3.07
Cd+Zn	13.55 ± 1.13 **	12.98 ± 0.64	12.83 ± 1.07 *	19.36 ± 2.84 **

Mean ± Standard Error. n = 10. Paired-sample *t*-test: *, *p* < 0.05; **, *p* < 0.01.

## References

[1] Zhang H., Tian Y., Wang L., Zhang L., Dai L. (2013). Ecophysiological characteristics and biogas production of cadmium-contaminated crops. Bioresour. Technol..

[2] Mcgrath S.P., Zhao J., Lombi E. (2002). Phytoremediation of metals, metalloids, and radionuclides. Adv. Agron..

[3] Yue Z.B., Yu H.Q., Wang Z.L. (2007). Anaerobic digestion of cattail with rumen culture in the presence of heavy metals. Bioresour. Technol..

[4] Verma V.K., Singh Y.P., Rai J.P.N. (2007). Biogas production from plant biomass used for phytoremediation of industrial wastes. Bioresour. Technol..

[5] Cao Z., Wang S., Wang T., Chang Z., Shen Z., Chen Y. (2015). Using contaminated plants involved in phytoremediation for anaerobic digestion. Int. J. Phytoremediat..

[6] Pobeheim H., Munk B., Johansson J., Guebitz G.M. (2010). Influence of trace elements on methane formation from a synthetic model substrate for maize silage. Bioresour. Technol..

[7] Zandvoort M.H., Van Hullebusch E.D., Fermoso F.G., Lens P.N.L. (2006). Trace metals in anaerobic granular sludge reactors: Bioavailability and dosing strategies. Eng. Life Sci..

[8] Ashekuzzaman S.M., Poulsen T.G. (2010). Optimizing feed composition for improved methane yield during anaerobic digestion of cow manure based waste mixtures. Bioresour. Technol..

[9] Chachkhiani M., Dabert P., Abzianidz T., Partskhaladze G., Tsiklauri L., Dudauri T., Godon J.J. (2004). 16S rDNA characterisation of bacterial and archaeal communities during start-up of anaerobic thermophilic digestion of cattle manure. Bioresour. Technol..

[10] Tian Y., Zhang H., Zheng L., Li S., Hao H., Yin M., Cao Y., Huang H. (2019). Process analysis of anaerobic fermentation exposure to metal mixtures. Int. J. Environ. Res. Public Health.

[11] Mudhoo A., Kumar S. (2013). Effects of heavy metals as stress factors on anaerobic digestion processes and biogas production from biomass. Int. J. Environ. Sci. Technol..

[12] Glass J.B., Orphan V.J. (2012). Trace metal requirements for microbial enzymes involved in the production and consumption of methane and nitrous oxide. Front. Microbiol..

[13] Manyiloh C.E., Mamphweli S.N., Meyer E.L., Okoh A.I., Makaka G., Simon M. (2013). Microbial anaerobic digestion (bio-digesters) as an approach to the decontamination of animal wastes in pollution control and the generation of renewable energy. Int. J. Environ. Res. Public Health.

[14] Gallego S.M., Pena L.B., Barcia R.A., Azpilicueta C.E., Iannone M.F., Rosales E.P., Zawoznik M.S., Groppa M.D., Benavides M.P. (2012). Unravelling cadmium toxicity and tolerance in plants: Insight into regulatory mechanisms. Environ. Exp. Bot..

[15] Jain S.K., Gujral G.S., Jha N.K., Vasudevan P. (1992). Production of biogas from *Azolla pinnata* R.Br and *Lemna minor* L: Effect of heavy metal contamination. Bioresour. Technol..

[16] Lin C.Y. (1992). Effect of heavy metals on volatile fatty acid degradation in anaerobic digestion. Water Res..

[17] Yu H.Q., Fang H.H. (2001). Inhibition by chromium and cadmium of anaerobic acidogenesis. Water Sci. Technol..

[18] Taherzadeh M.J., Karimi K. (2008). Pretreatment of lignocellulosic wastes to improve ethanol and biogas production: A review. Int. J. Mol. Sci..

[19] Šotnar M., Mareček J., Máchal P., Koutný T., Geršl M., Krčálová E., Korenko M. (2014). Biogas production of phytoremediation plants contaminated with cadmium. Научни Tpyдoве Pyceнския Университет.

[20] Elizabeth L.S., MGeovanni S.M., Viridiana H.J., Rodolfo G.C., Rafael M.S., Ricardo J.C. (2012). Activation of methanogenesis by cadmium in the marine archaeon *Methanosarcina acetivorans*. PLoS ONE.

[21] Hao H., Tian Y., Zhang H., Chai Y. (2017). Copper stressed anaerobic fermentation: Biogas properties, process stability, biodegradation and enzyme responses. Biodegradation.

[22] Sauer K., Thauer R.K. (2000). Methyl-coenzyme M formation in methanogenic archaea. Involvement of zinc in coenzyme M activation. Eur. J. Biochem..

[23] Bożym M., Florczak I., Zdanowska P., Wojdalski J., Klimkiewicz M. (2015). An analysis of metal concentrations in food wastes for biogas production. Renew. Energy.

[24] Zhang H., Han X., Tian Y., Li Y., Yang K., Hao H., Chai Y., Xu X. (2018). Process analysis of anaerobic fermentation of *Phragmites* australis straw and cow manure exposing to elevated chromium (VI) concentrations. J. Environ. Manag..

[25] Lei Z., Chen J., Zhang Z., Sugiura N. (2010). Methane production from rice straw with acclimated anaerobic sludge: Effect of phosphate supplementation. Bioresour. Technol..

[26] Lao J., Chen X., Qi M., Ji B., Lao J. (1988). Soil Agrochemical Analysis Manual.

[27] Wei Y., Van Houten R.T., Borger A.R., Eikelboom D.H., Fan Y. (2003). Minimization of excess sludge production for biological wastewater treatment. Water Res..

[28] Wei F. (2002). Monitoring and Analysis Methods of Water and Wastewater.

[29] Su Y. (2011). Biogas Fermentation Detection Technology.

[30] Angenent L.T., Sung S., Raskin L. (2002). Methanogenic population dynamics during startup of a full-scale anaerobic sequencing batch reactor treating swine waste. Water Res..

[31] Lo H.M., Chiang C.F., Tsao H.C., Pai T.Y., Liu M.H., Kurniawan T.A., Chao K.P., Liou C.T., Lin K.C., Chang C.Y. (2012). Effects of spiked metals on the MSW anaerobic digestion. Waste Manag. Res..

[32] Tian Y., Zhang H., Chai Y., Wang L., Mi X., Zhang L., Ware M.A. (2016). Biogas properties and enzymatic analysis during anaerobic fermentation of *Phragmites* australis straw and cow dung: Influence of nickel chloride supplement. Biodegradation.

[33] Zhai N., Zhang T., Yin D., Yang G., Wang X., Ren G., Feng Y. (2015). Effect of initial pH on anaerobic co-digestion of kitchen waste and cow manure. Waste Manag..

[34] Liu A., Xu S., Lu C., Peng P., Zhang Y., Feng D., Liu Y. (2014). Anaerobic fermentation by aquatic product wastes and other auxiliary materials. Clean Technol. Environ. Policy.

[35] Lee S.J. (2008). Relationship between Oxidation Reduction Potential (ORP) and Volatile Fatty Acid (VFA) Production in the Acid-Phase Anaerobic Digestion Process. Master’s Thesis.

[36] Zhang H., Tian Y., Wang L., Mi X., Chai Y. (2016). Effect of ferrous chloride on biogas production and enzymatic activities during anaerobic fermentation of cow dung and *Phragmites* straw. Biodegradation.

[37] Sawayama S., Tada C., Tsukahara K., Yagishita T. (2004). Effect of ammonium addition on methanogenic community in a fluidized bed anaerobic digestion. J. Biosci. Bioeng..

[38] Pokój T., Bułkowska K., Gusiatin Z.M., Klimiuk E., Jankowski K.J. (2015). Semi-continuous anaerobic digestion of different silage crops: VFAs formation, methane yield from fiber and non-fiber components and digestate composition. Bioresour. Technol..

[39] Béguin P., Aubert J.P. (1993). The biological degradation of cellulose. FEMS Microbiol. Rev..

[40] Deng S.P., Tabatabai M.A. (1995). Cellulase activity of soils: Effect of trace elements. Soil Biol. Biochem..

[41] Jaenchen R., Schönheit P., Thauer R.K. (1984). Studies on the biosynthesis of coenzyme F_420_ in methanogenic bacteria. Arch. Microbiol..

[42] Eirich L.D., Vogels G.D., Wolfe R.S. (1978). Proposed structure for coenzyme F_420_ from *Methanobacterium*. Biochemistry.

[43] Xi Y., Chang Z., Ye X., Xu R., Du J., Chen G. (2014). Methane production from wheat straw with anaerobic sludge by heme supplementation. Bioresour. Technol..

[44] Kretsinger R.H., Uversky V.N., Permyakov E.A. (2013). Encyclopedia of Metalloproteins.

[45] Fosses A., Maté M., Franche N., Liu N., Denis Y., Borne R., De Philip P., Fierobe H.P., Perret S. (2017). A seven-gene cluster in *Ruminiclostridium cellulolyticum* is essential for signalization, uptake and catabolism of the degradation products of cellulose hydrolysis. Biotechnol. Biofuels.

[46] Niu L., Song L., Liu X., Dong X. (2009). *Tepidimicrobium xylanilyticum* sp. nov., an anaerobic xylanolytic bacterium, and emended description of the genus *Tepidimicrobium*. Int. J. Syst. Evol. Microbiol..

[47] Liu T., Ahn H., Sun W., McGuinness L.R., Kerkhof L.J., Häggblom M.M. (2016). Identification of a *Ruminococcaceae* Species as the Methyl *tert*-Butyl Ether (MTBE) Degrading Bacterium in a Methanogenic Consortium. Environ. Sci. Technol..

[48] Weiss G.A., Hennet T. (2012). The role of milk sialyllactose inintestinal bacterial colonization. Adv. Nutr..

[49] Cibis K.G., Wibberg D., Maus I., Schlüter A., Pühler A., Winkler A., König H., Stolze Y. (2015). Complete genome sequence of the strain *Defluviitoga tunisiensis* L3, isolated from a thermophilic, production-scale biogas plant. J. Biotechnol..

[50] Speece R.E. (1996). Anaerobic biotechnology for industrial wastewater treatment. Environ. Sci. Technol..

[51] Balch W., Fox G.E., Magrum L.J., Woese C.R., Wolfe R.S. (1979). Methanogens: Reevaluation of a unique biological group. Microbiol. Rev..

